# Adverse cardiovascular effect following gonadotropin-releasing hormone antagonist versus GnRH agonist for prostate cancer treatment: A systematic review and meta-analysis

**DOI:** 10.3389/fendo.2023.1157857

**Published:** 2023-03-31

**Authors:** Li Gu, Xurui Li, Wentao Liu

**Affiliations:** ^1^ Department of Gastroenterology, The Second Xiangya Hospital, Central South University, Changsha, Hunan, China; ^2^ Department of Urology, The Second Xiangya Hospital, Central South University, Changsha, Hunan, China

**Keywords:** GnRH, antagonist, agonist, cardiovascular event, prostate cancer

## Abstract

**Background:**

Androgen deprivation therapy is the mainstay of medical treatment for prostate cancer (Pca); however, it is associated with an increased risk of adverse cardiovascular (CV) events and death. To date, CV death has been the leading noncancer cause of death in Pca patients. Both GnRH antagonists (an emerging class of drugs) and GnRH agonists (most frequently prescribed) are efficacious against Pca. However, the adverse effects, especially the adverse CV effect between them remain unclear.

**Methods:**

Through a literature search using MEDLINE, EMBASE and the Cochrane Library, all available studies comparing the safety of CV risk between GnRH antagonists and GnRH agonists in Pca patients were extracted. Comparisons of outcomes of interest between these two classes of drugs were calculated using the risk ratio (RR). Subgroup analyses were performed depending on the study design and preexisting CV disease at baseline.

**Results:**

Nine randomized controlled clinical trials (RCTs) and five real-world observational studies comprising 62160 Pca patients were included in our meta-analysis. Patients receiving GnRH antagonists experienced fewer CV events (RR: 0.66, 95% CI:0.53-0.82, P<0.001), CV death (RR:0.4, 95% CI: 0.24-0.67, P<0.001) and myocardial infarctions (RR: 0.71, 95% CI: 0.52-0.96, P=0.03). No difference was found in the incidence of stroke and heart failure. Moreover, GnRH antagonists were associated with fewer CV events in patients with preexisting CV disease but not in those without preexisting CV disease in the RCT series.

**Conclusion:**

GnRH antagonists appear to offer favorable safety in terms of adverse CV events and CV death compared with GnRH agonists among men diagnosed with Pca, especially those who had established CV disease at baseline.

**Systematic review registration:**

https://inplasy.com/inplasy-2023-2-0009/, identifier INPLASY202320009.

## Introduction

Androgen deprivation therapy (ADT) is the mainstay of medical treatment for prostate cancer (Pca). It is mainly used to treat men with metastatic Pca, or as neoadjuvant/adjuvant therapy to men with high risk locally advanced Pca, accounting for approximately 40% Pca patients (1). Despite the fact that ADT improves the lives of Pca patients in terms of quality of life and cancer mortality, it also increases their likelihood of experiencing adverse events (2). Among these, cardiovascular(CV) disease is the major adverse event and has been the leading noncancer cause of death in Pca patients (3). Thus, any effort to minimize it is of great value.

Among the different types of ADT, gonadotropin-releasing hormone (GnRH) agonists, such as leuprorelin and goserelin, have been the most frequently prescribed ADT drugs during the past few years. Through continuous stimulation of GnRH receptors at the anterior pituitary, GnRH agonists “indirectly” reduce the level of testosterone, along with a transient elevation of gonadotropin and testosterone at the initial phase (4). Unlike GnRH agonists, GnRH antagonists (such as degarelix), as an emerging class of drugs, “directly” block GnRH receptors, leading to a rapid reduction in testosterone levels without the flare of gonadotropin and testosterone (4).

Numerous studies have been designed to compare GnRH antagonists with GnRH agonists in the setting of oncological outcomes and adverse events. In 2021, Abufaraj et al. (5) conducted a meta-analysis of eight randomized controlled clinical trials (RCTs) and concluded that GnRH antagonists and GnRH agonists are equivalently efficacious against Pca, however, they have different adverse effects. Among six RCTs with 2318 Pca patients in which CV events were reported, users of GnRH antagonists had a lower risk of adverse CV events than users of GnRH agonists. In contrast, another meta-analysis published in the same year comprising four RCTs (2408 patients) and two real-world observational studies (29349 patients) indicated that no difference was found between usage of GnRH antagonists and GnRH agonists regarding adverse CV events in either RCTs or real-world data (6). These findings indicated that there is still debate from clinical trials and real-world data as to whether GnRH antagonists causes fewer CV adverse events than GnRH agonists. Additionally, CV related death and some particular CV events (myocardial infarction, etc.) have never been described in the aforementioned meta-analyses. Furthermore, the potential association between preexisting CV disease and the adverse CV effect of ADT is still unclear.

To fully address the above issue, we performed a meta-analysis including all available RCTs and real-world studies. Notably, in addition to CV events, we also considered CV related death, some specific CV events and preexisting CV disease at baseline.

## Methods

### Search strategy and study selection

Following the guidelines established by the Preferred Reporting Items for Systematic Reviews and Meta-Analyses (PRISMA) (7), a comprehensive review was conducted. This study is registered at the INPLASY register (INPLASY202320009). For this purpose, we searched MEDLINE, EMBASE, and the Cochrane Library up to November 2022 that met our criteria for inclusion. The search terms were (‘‘gonadotropin-releasing hormone antagonist” OR ‘‘antagonist” OR “degarelix”) AND (‘‘gonadotropin-releasing hormone agonist” OR ‘‘agonist” OR ‘‘goserelin” OR “leuprolide” OR “triptorelin” OR “histrelin”) AND (“prostate”). There were linguistic constraints in English. The references of retrieved articles were also searched for additional studies. The following requirements were satisfied by the chosen studies: (1) randomized controlled trials, prospective or retrospective cohort study; (2) compared GnRH antagonist with GnRH agonist in patients diagnosed with prostate cancer; (3) reported any cardiovascular events or cardiovascular death in both arms; (4) letter to the editor, reviews, case-series and case-reports were not considered, and (5) in the event when studies focused on the same clinical trial or real-world database, the study providing more relevant information was included. Additional information from a *post hoc* analysis (8) comprising individual patient data from six RCTs was also extracted.

### Data extraction and risk of bias

Two reviewers meticulously and independently retrieved data from relevant research, which may include study demographics, baseline characteristics of patients, adverse CV events and CV death. Study demographics extracted included first author name, year, patient’s country, study design, drug types and dosage, sample size, and follow-up. Baseline characteristics of patients included age, testosterone levels, preexisting CV disease, hypertension and diabetes. Adverse CV events were defined when any of the following occurred: acute coronary syndromes, myocardial infarction, heart failure, cardiac arrest, stroke, hypertension, arterial embolic and thrombotic events. In addition, detailed data on CV events in Pca patients with or without preexisting CV disease were separately collected when reported. The quality of individual included studies was independently assessed based on the Downs and Black tool (9) by two reviewers. The score of the Downs and Black tool ranges from 0 to 28. All disagreements were resolved through discussions with the third investigator.

### Statistical analysis

The risk ratio (RR), along with the 95% confidence interval (95% CI), was used to compare the effects of GnRH antagonists with respect to the outcomes of interest in each article. For studies with incomparable follow-up between GnRH antagonist and agonist users (10–14), the hazard ratio (HR) was extracted. RRs or HRs of each study were used for subsequent pooled analysis. Statistical significance was assumed at a p value < 0.05. Chi-square-based Q tests and I ^2^ statistics were used to determine whether there was significant heterogeneity across studies. In cases where there was high heterogeneity, as indicated by an I ^2^ value > 50% and P value < 0.05, the pooled effect was determined using a random-effects model (the DerSimonian and Laird method). Otherwise, we conducted the meta-analysis using a fixed-effects approach (the Mantel-Haenszel technique) (15). To investigate the causes of the heterogeneity, subgroup analyses were carried out. We checked for possible instances of publication bias using both Begg’s funnel plot and Egger’s test. Review Manager version 5.4 was used to conduct all statistical studies.

## Results

### Study process

**Figure 1:**
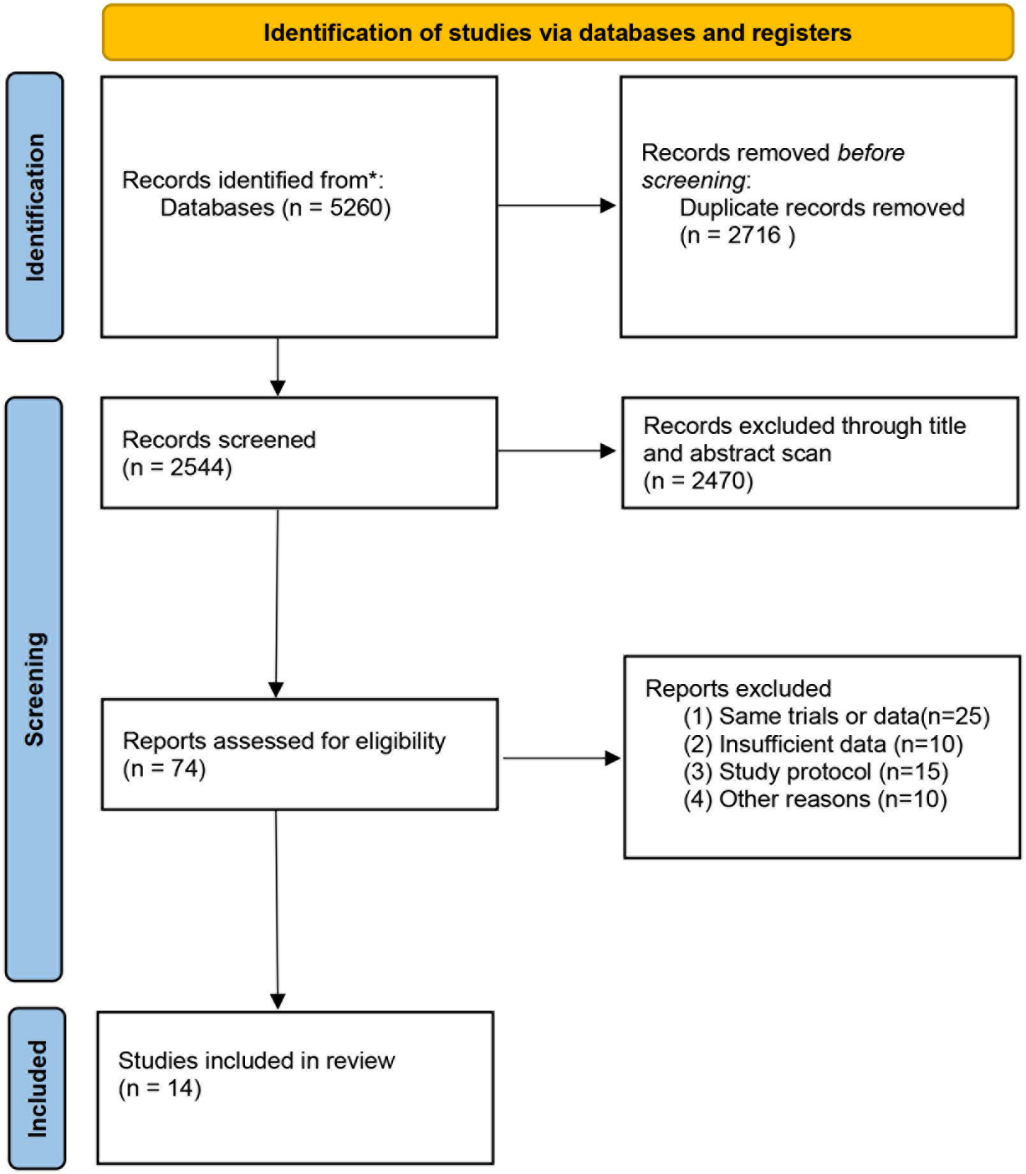
Flow diagram of selection of eligible studies.

**Figure 1** is a flowchart depicting the processes used to choose relevant literature. Our literature search yielded a total of 5260 articles. After excluding duplicates and articles that did not meet the inclusion criteria based on the title and abstract scan, 74 articles were included for further full-text evaluation. Then, 60 articles were excluded due to the same database or trial, absence of reporting cardiovascular events and others, leaving 14 articles comparing the GnRH antagonist versus GnRH agonist for our meta-analysis.

### Characteristics of the included studies and patients

In total, fourteen studies with 62160 Pca patients were included. Nine of these investigations (16–24) were RCTs, both of which were phase II or phase III clinical trials (1b level of evidence), comprising 1912 Pca patients treated with GnRH antagonists and 1221 Pca patients treated with GnRH agonists. The remaining five studies (10–14) were retrospective real-world observational studies (2b level of evidence) from French, Italian, Taiwan, UK or global population database, comprising 4210 GnRH antagonist recipients and 54817 GnRH agonist recipients (**Table 1**). All eligible studies administered degarelix as a GnRH antagonist, while three, five and six studies administered leuprorelin, goserelin and heterogeneous drugs (one of leuprorelin, goserelin triptorelin and histrelin) as GnRH agonists, respectively. According to the quality ratings, the majority of the RCTs and real-world studies were considered to be of a high and moderate standard, respectively. In the real-world data ranging from 2010 to 2018, 7.1% of patients were treated with GnRH antagonists as compared to 92.9% of patients treated with GnRH agonists.

**Table 1:** Characteristics and quality score of included studies.

Author	Year	Trials, country	Design, LOE	Drugs	Numbers	GnRH antagonist dosage	GnRH agonist dosage	Follow-up (mo)	Quality score
Klotz (23)	2008	CS21, Global	RCT,1b	Degarelix Leuprolide	409 201	Degarelix 80 mg monthly or 160 mg monthly	Leuprolide 7.5 mg monthly	12	25
Axcrona (22)	2012	CS31, Scandinavia	RCT,1b	Degarelix Goserelin	82 97	Degarelix 80 mg monthly	Goserelin 3.6 mg monthly	3	25
Tombal (21)	2012	CS35, Global	RCT,1b	Degarelix Goserelin	565 282	Degarelix 480 mg 3-monthly	Goserelin 3.6 mg monthly	12	21
Anderson (20)	2013	CS28, UK	RCT,1b	Degarelix Goserelin	27 13	Degarelix 80 mg monthly	Goserelin 3.6 mg monthly	3	21
Mason (19)	2013	CS30, USA and Europe	RCT,1b	Degarelix Goserelin	181 64	Degarelix 80 mg monthly	Goserelin 3.6 mg monthly	3	22
Higano (18)	2015	CS37, USA	RCT,1b	Degarelix Leuprolide	225 178	Degarelix 80 mg monthly	Leuprolide 7.5 mg monthly	14	24
Scailteux (14)	2017	Real-World, French	R,2b	Degarelix Agonist&	1273 24846	NR	NR	16	14
Margel (17)	2019	Israel	RCT,1b	Degarelix Agonist	41 39	Degarelix 80 mg monthly	NR	12	26
Cone (13)	2020	Real-World, Global	R,2b	Degarelix Agonist	1606 10504	NR	NR	>12	16
Perrone (12)	2020	Real-World, Italian	R,2b	Degarelix Agonist	530 9070	NR	NR	74	15
Sun (24)	2020	China	RCT,1b	Degarelix Goserelin	142 141	Degarelix 80 mg monthly	Goserelin 3.6 mg monthly	12	24
Chen (11)	2021	Real-World, Taiwan	R,2b	Degarelix Agonist	666* 1332	NR	NR	36	18
Davey (10)	2021	Real-World, UK	R,2b	Degarelix Agonist	101 8980	NR	NR	8	16
Lopes (16)	2021	NCT02663908, Global	RCT,1b	Degarelix Leuprolide	275 269	Degarelix 80 mg monthly	Leuprolide 22.5 mg 3-monthly	12	25

LOE, level of evidence; R, retrospective. Agonist& means usage of one of leuprolide, goserelin, triptorelin or histrelin. RCT, randomized controlled trial; NR, none reported. * Propensity Score Matched from 708 and 10619 patients treated with Degarelix and Agonist.

At baseline, no differences were detected between GnRH antagonist users and GnRH agonist users in terms of age, testosterone levels, preexisting CV disease, hypertension and diabetes (**Supplementary Figure 1**, all p >0.05), indicating comparable baseline characteristics of the included patients. Similarly, subgroup analysis showed that no differences were detected in terms of the above five parameters in either RCTs or real-world data (**Supplementary Figure 1**, all p >0.05).

### Adverse cardiovascular events

**Figure 2:**
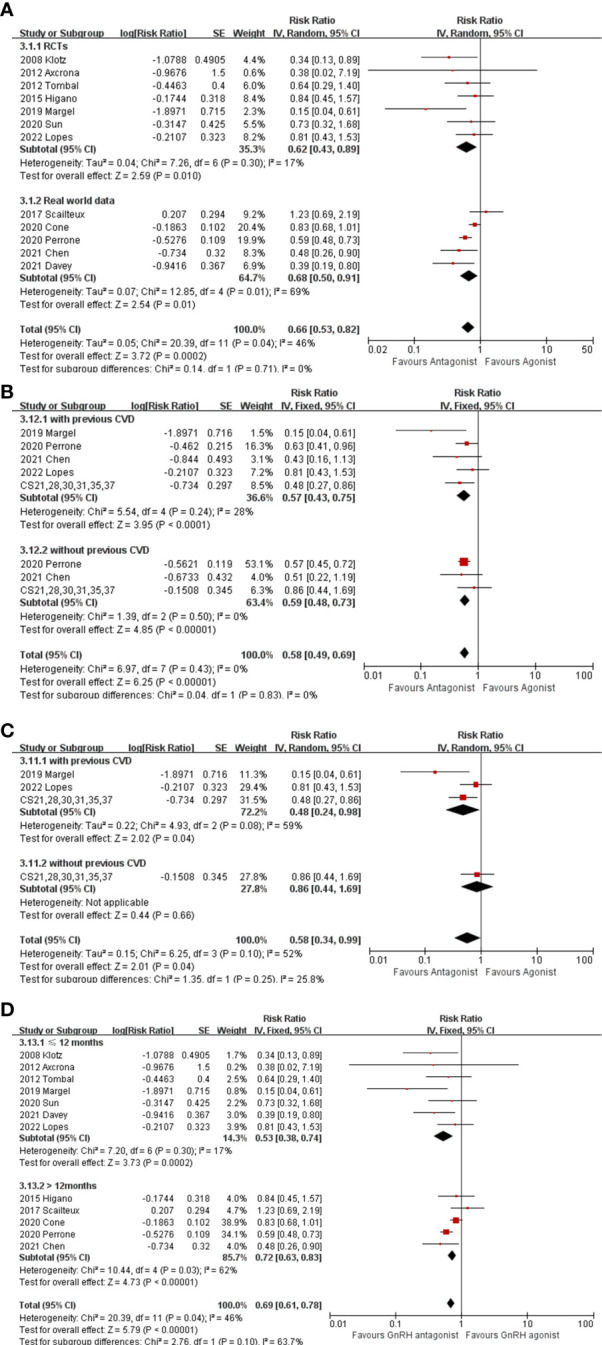
**(A)** The forest plot of CV events in RCTs and real-world data. **(B)** The forest plot of CV events stratified with preexisting CV disease. **(C)** The forest plot of CV events stratified with preexisting CV disease in RCTs. **(D)** The forest plot of CV events stratified with follow-up period. The boxes, lines and rhombus represent RR (or HR), 95% CI of each study and the pooled RR along with 95% CI, respectively.

Among 6122 GnRH antagonist and 56038 GnRH agonist recipients, 252 (4.1%) and 3628 (6.4%) CV events were reported, respectively. No cardiovascular events were recorded in two trials (19, 20) with a maximum follow-up of 3 months, thus their results were not used for the pooled RR estimate. As displayed in **Figure 2A**, the pooled outcomes revealed that GnRH antagonists were associate with fewer CV events compared with GnRH agonist (RR: 0.66, 95% CI:0.53-0.82, P<0.001, I^2 =^ 46%). In the subgroup analysis, GnRH antagonists were associate with fewer CV events than GnRH agonists in both RCTs (**Figure 2A**, RR: 0.62, 95% CI: 0.43-0.89, P=0.01, I^2 =^ 17%) and real-world data (**Figure 2A**, RR: 0.68, 95% CI: 0.50-0.91, P=0.01, I^2 =^ 69%).

Two studies (16, 17) focused only on patients with preexisting CV disease, and eight studies (18–23) provided individual information on patients who had or had not a history of CV disease, hence; these ten studies were extracted. In addition, individual patient data from the six aforementioned RCTs (18–23) were included in a *post hoc* analysis by Abertsen et al. (8) and were extracted for subsequent pooled analysis. Compared with GnRH agonist users, there were significantly fewer CV events experienced by GnRH antagonist users among either men with preexisting CV disease (**Figure 2B**, RR: 0.57, 95% CI: 0.43-0.75, P<0.001, I^2 =^ 28%) or men without preexisting CV disease (**Figure 2B**, RR: 0.59,95% CI: 0.48-0.73, P<0.001, I^2 =^ 0%). However, when only considering RCTs, no differences in the incidence of CV events were found between GnRH antagonists and GnRH agonists among men without a history of CV disease (**Figure 2C**, RR: 0.86, 95% CI: 0.44-1.69, P=0.66), except for among men with a history of CV disease (**Figure 2C**, RR: 0.48, 95% CI: 0.24-0.98, P=0.04, I^2 =^ 59%). Additionally, subgroup analysis depending on time of follow-up (>12 months or <12 months) was performed (**Figure 2D**). The results showed that GnRH antagonist recipients experienced less CV even during either the short-term (RR: 0.53, 95% CI: 0.38-0.74, P<0.001, I^2 =^ 17%) or the relatively long-term follow-up period (RR: 0.72, 95% CI: 0.63-0.83, P<0.001, I^2 =^ 62%).

### Cardiovascular death

**Figure 3:**
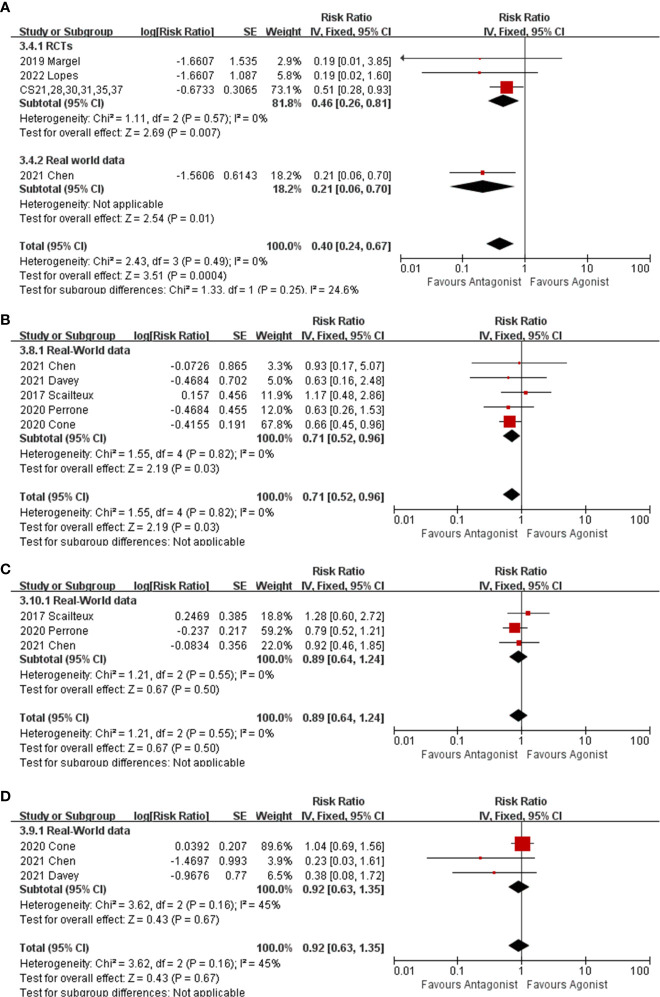
The forest plot of CV death **(A)**, myocardial infarction **(B)**, stoke **(C)** and heart failure **(D)**. The boxes, lines and rhombus represent RR(or HR), 95% CI of each study and the pooled RR along with 95% CI, respectively.

Nine studies comprising 2473 GnRH antagonist and 2477 GnRH agonist recipients reported CV death, among which 23 (0.9%) and 50 (2%) CV deaths occurred, respectively. The RR of CV death for GnRH antagonists as compared with GnRH agonists was 0.4 (95% CI: 0.24-0.67, P<0.001, I^2 =^ 0%, **Figure 3A**). The subgroup analysis also showed significantly fewer CV deaths among GnRH antagonist recipients in RCTs (**Figure 3A**, RR: 0.46, 95% CI: 0.26-0.81, P=0.007, I^2 =^ 0%) and real-world data (**Figure 3A**, RR: 0.21,95% CI: 0.06-0.70, P=0.01).

### Specific cardiovascular event

Five real-world cohorts (10–14) reported individual myocardial infarctions and indicated that GnRH antagonist recipients experienced fewer myocardial infarctions than GnRH agonist recipients (**Figure 3B**, RR:0.71,95% CI: 0.52-0.96, P=0.03,I^2^ = 0%). There were no differences in the incidence of stroke and heart failure between patients receiving GnRH antagonist and GnRH agonist (**Figure 3C**, stroke, RR: 0.89, 95% CI: 0.64-0.1.24, P=0.55, I^2^ = 0%) (**Figure 3D**, heart failure, RR: 0.92, 95% CI: 0.63-1.35, P=0.67, I^2^ = 45%).

### Publication bias

There was no discernible asymmetry in Begg’s funnel plot of CV events (**Supplementary Figure 1**) and other results. Moreover, Egger’s test p values for each outcome were >0.05, also indicating no substantial publication bias.

## Discussion

CV disease is a major adverse event associated with ADT. To date, CV death has been the leading noncancer cause of death in Pca patients. Thus, any effort to minimize it is of great value. The major findings from our meta-analysis are that Pca patients treated with GnRH antagonists are associated with lower occurrences of CV events, CV death and myocardial infarction than those treated with GnRH agonists. In our results, the relative risk reduction appears to be as high as 35%, 60% and 30% in terms of CV events, CV death and myocardial infarction, respectively. In contrast to the aforementioned meta-analyses, the benefit of GnRH antagonists remains consistent in both clinical trials and real-world data in our meta-analysis. In general, RCT samples are highly selected, while real-world data are complementary to RCT data (25). Gathering the evidence from RCTs and real-world studies would be helpful to gain a more complete picture of the advantages and disadvantages of medications as they are used in practice (26).

Notably, five national databases ranging from 2010 to 2018 (10–14) in the present study showed that only 7.1% of patients were treated with GnRH antagonists as compared to 92.9% of patients treated with GnRH agonists. Indeed, these two classes of drugs are thought to be equally efficacious against prostate cancer. It seems that urologists and patients appear to be unaware of the difference in adverse events between these two classes of drugs and prefer to choose the cheaper option (GnRH agonist).

It is still debatable whether there is a potential association between preexisting cardiac disease and the adverse effect of ADT, despite the large number of studies reporting the results of men taking ADT. According to the study by Nanda et al. (27), an elevated risk of CV disease was only seen in males who already had previous cardiovascular disease. Abertsen et al. (8) found in a *post hoc* study that males who received a GnRH agonist were twice as likely to have a CV incident as those who received an antagonist. Among males who did not already have CV disease, there was no change in the rate at which CV events occurred. The absolute risk reduction of CV events in males who had a history of CV disease and males who had no history CV disease at baseline was 4.9% and 0.4%, respectively. Our meta-analysis indicated that GnRH antagonists reduced the risk of CV events among either men with a history of CV disease or those without a history of CV disease. However, when only considering RCTs, no differences in the incidence of CV events were found between these two classes of drugs in men without preexisting CV disease. In summary, the advantage of GnRH antagonists regarding CV risk seems to be conspicuous in men with preexisting CV disease.

Additionally, some major risk factors could affect CV events and death, including older age, hypertension (28), hypertriglyceridemia (29), insulin resistance (28), and alcohol intake (30). In our meta-analysis, the baseline characteristics in terms of age, testosterone levels preexisting CV disease, hypertension and diabetes were comparable between groups, indicating that the influence of these risk factors on the final results is minute. Prostate cancer typically occurs in elderly individuals, who are more likely to have CV risk factors. According to a Longitudinal Prostate Cancer (RADICAL PC) study, two-thirds of a cohort of 2492 patients with Pca are at high CV risk and 22% have established CV disease (31). Together with adverse effects of ADT on the cardiovascular system, quite a few patients with Pca may experience future CV adverse outcomes. Therefore, it is reasonable to believe that GnRH antagonists, instead of GnRH agonists, should be recommended to Pca patients with any risk factors for CV disease. However, future well-designed studies are still needed to evaluate the association between these risk factors and ADT drugs.

However, to be completely described are the mechanisms by which GnRH agonists and antagonists can confer differential risks of CV events. The development and breakage of atherosclerotic plaques are the leading causes of CV events such heart attacks, strokes, and other cardiovascular-related hospitalizations (32). GnRH antagonist treatment in a mouse model of atherosclerotic vascular disease resulted in significantly lower levels of atherosclerotic plaque when compared to GnRH agonist treatment (33). Therapy with leuprolide, but not degarelix, enhanced regions of necrosis in the plaques in another study examining the effects of ADT on preexisting plaque in apolipoprotein E-deficient animals (34). Plaques susceptible to rupture have a substantial core of lipids and necrotic material surrounded by a thin, inflammatory fibrous covering (35). Matrix metalloproteinases associated with macrophages are responsible for degrading the cap connective tissue, leading to rupture (36). On the other hand, T cells express GnRH receptors, and it has been demonstrated that activation of GnRH receptors by GnRH agonists, but not GnRH antagonists, stimulates T-cell expansion and differentiation into the T-helper (Th1) phenotype (11) making it a key activator of macrophages. Enhanced invasiveness and expression of matrix metalloproteinases‐9 are both indicators of a macrophage that has been activated, which could make the atherosclerotic plaque more vulnerable to rupturing by weakening its fibrous cap (37).

There are some limitations that should be taken into account. First, the bulk of the outcomes demonstrated moderate and high heterogeneity, which might be attributed to differences in patient population, study design, follow-up period and reporting technique of end point between real-world observational research. There was a significant decrease in diversity among the RCT subgroups. Second, some factors (e.g. drug dosage and drug treatment duration) affecting the final results were not well described in real world cohort studies, which potentially resulted in bias. Additionally, the follow-up period in the majority of included studies was short. It is currently unknown what effect these two classes of ADT drugs will have on the long-term impact of men with Pca, especially those without a history of CV disease. Furthermore, as the bulk of the included RCTs were not pertinently intended to evaluate CV events, there is a possibility of underreporting CV occurrences. Despite these limitations, ours is the most comprehensive meta-analysis on the CV safety of the two classes of ADT drugs, and is the first to analyze CV death, specific CV events and the internal association between preexisting CV disease and usage of these two classes of ADT drugs in patients with Pca.

In conclusion, GnRH antagonists appear to offer favorable safety in terms of adverse CV events and CV death compared with GnRH agonists in men diagnosed with Pca, especially those at high risk of CV disease.

## Data availability statement

The original contributions presented in the study are included in the article/**Supplementary Material**. Further inquiries can be directed to the corresponding author.

## Author contributions

LG: Statistical analysis, data extraction, revised manuscripts. XL: Statistical analysis, data extraction. WL: Study design, statistical analysis, data extraction, wrote the paper. All authors contributed to the article and approved the submitted version.

## Supplementary material

Supplementary Figure 1 The forest plot of age **(A)**, testosterone levels **(B)**, preexisting CV disease **(C)** hypertension **(D)** and diabetes I. Begg’s funnel plot of CV events **(F)**. The boxes, lines and rhombus represent RR(or HR), 95% CI of each study and the pooled RR along with 95% CI, respectively.
